# Cardiologists' perspective on termination of pacemaker therapy–an anonymous survey among cardiologists in Germany

**DOI:** 10.1007/s00392-024-02525-z

**Published:** 2024-09-02

**Authors:** Irene Portig, Elena Hofacker, Philipp Sommer, Christian Volberg, Carola Seifart

**Affiliations:** 1 Research Group Medical Ethics, Faculty of Medicine, Philipps University of Marburg, Baldingerstraße, 35043 Marburg, Germany; 2 Clinic for Electrophysiology, Herz- und Diabeteszentrum NRW, University Hospital of Ruhr University Bochum, Bad Oeynhausen, Germany; 3 Department of Anaesthesiology and Intensive Care Medicine, Philipps University of Marburg, Baldingerstraße, 35043 Marburg, Germany

**Keywords:** Cardiology, Pacemaker withdrawal, Pacemaker dependency, Medical ethics, End-of-life care

## Abstract

**Background:**

The patient’s right to refuse pacemaker therapy is mentioned in the relevant European consensus statement but additional information is only available on deactivation of implantable cardioverter deactivator and not on other cardiac implantable electronic devices such as pacemakers. Therefore, we were interested in opinions, concerns and attitudes of cardiologists, who are the primary contact persons for such requests, since the number of patients asking for withdrawal of pacemaker therapy is likely to increase leaving cardiologists and healthcare professionals with a difficult medical but also ethical problem.

**Methods:**

An anonymous questionnaire was sent to all German cardiology departments (*N* = 288).

**Results:**

48% of cardiology departments responded by sending back 247 completed questionnaires. Most participating cardiologists were experienced when considering the duration of their professional activity. Almost all of the respondents regularly perform check-ups of pacemakers. The majority of cardiologists answering our questionnaire were prepared to deactivate a pacemaker upon patients’ request, and have done so. In pacemaker dependency, however, the willingness to withdraw decreases, even if death is imminent, for fear of causing distressing symptoms, sense of being responsible for patients possible immediate death, or fear of legal consequences.

**Conclusions:**

The survey could clearly show that uncertainties remain among cardiologists dealing with a patient's wish for withdrawal, especially in cases of pacemaker dependency. We suggest that official statements of cardiologic societies in Europe are issued to clarify ethical, legal and practical aspects of pacemaker withdrawal.

**Trial registration:**

Registered in the German Clinical Trials Register (DRKS00026168) on 30.08.2021.

**Graphical Abstract:**

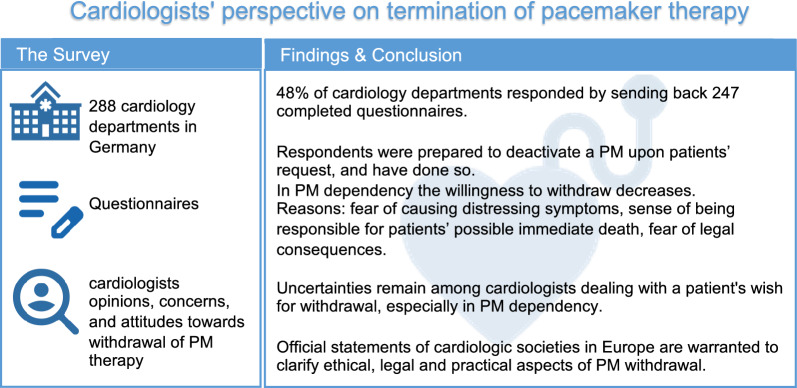

## Introduction

The US American Heart Rhythm Association issued expert consensus statements affirming the ethical and legal permissibility of cardiac implantable electronic devices (CIED) deactivation, even if the patient is not terminally ill [1]. This statement was developed in collaboration with members of the European Heart Rhythm Association (EHRA). The equivalent European consensus statement focuses on the deactivation of implantable cardioverter defibrillators (ICDs) in dying patients [2]. It points out, that less agreement exists in Europe for PM deactivation. In Germany, an equivalent statement was published in 2017 regarding the withdrawal of ICD but not pacemaker (PM) therapy [3]. The legal situation regarding PM withdrawal in Germany is still a matter of debate and has not been finally clarified [4].

The consequences of withdrawal of PM therapy vary depending on indication: Sinunodal disease (SND) and higher degree atrioventricular block (AVB) are the most common forms of bradyarrhythmia, where permanent PMs provide effective treatment [5, 6]. SND is not a life-threatening condition, the benefit of PM therapy is essentially to alleviate symptoms (syncope leading to injuries from falls, breathlessness, or fatigue) [7]. By deactivation of PM therapy an effective treatment will be discontinued. In high-grade AVB, PM therapy is known to prevent sudden cardiac death in addition to the aforementioned improvement of quality of life (reviewed in [6]). In these cases, deactivation of PM therapy will probably lead to worsening of symptoms and sudden cardiac death may occur.

In some patients, PM dependency can be detected. In PM dependency an intrinsic rhythm ≥ 30 bpm is absent after lowering the pacing rate to 30 bpm for at least 10 s or after transient inhibition of pacing therapy. It must be noted, that a number of definitions for PM dependency exist and several authors have called for standardization of the term [8]. In PM dependency, PM therapy is thought to prolong life [1] and may prolong the dying process. After the withdrawal of PM therapy in PM-dependent patients an escape rhythm normally ensues. Depending on how quickly this happens, death, loss of conscience, organ failure due to prolonged asystole e.g. hypoxic ischemic encephalopathy, or (severe) symptoms of heart failure may follow [2, 9].

PM dependency is believed to occur only rarely. In a recent study, follow-up examinations of patients with PM could show, that 16% (131 out of 802) were found to be pacing-dependent [10]. Along with increasing numbers of PM implantations, PM dependency seems to occur more frequently in an aging population [10].

Management of PM therapy is at least in Germany usually in the hand of cardiologists. For the withdrawal of PM therapy, reprogramming of PM using special equipment is needed where each company has its own device. Apart from these technical issues, the request for termination of PM therapy, particularly in PM dependency, are part of end of life (EOL) decisions. Therefore, cardiologists are not only confronted with a difficult medical but also ethical problem.

Most healthcare professionals regard device deactivation in dying patients as allowing natural death, especially when intended to alleviate symptoms and not to hasten death [11–15]. However, in PM-dependent patients a number of physicians object to deactivating PMs, arguing that it can either lead to symptoms of heart failure or death [1, 16, 17].

The question of whether and how PM therapy can and should be withdrawn at the request of an actively dying patient can be a challenge. It can be even more difficult if the patient who requests discontinuation of PM therapy, is nearing EOL, but yet not actively dying, or not even terminally ill (these terms are used according to [18, 19]). Health care professionals including those without cardiologic expertise will in the future have to deal with these questions. We have therefore conducted a survey in Germany among cardiologists working in hospitals to learn more about their opinions, concerns, and attitudes towards withdrawal of PM therapy.

## Methods

In January 2022 paper versions of an anonymous questionnaire were sent to heads of all cardiology departments of hospitals (*N* = 288) listed in the German hospital registry allowing more than one questionnaire to be returned (physician-based analysis).

Our survey was conducted using a questionnaire with close-ended questions to capture participants’ experience with PM deactivation. In addition, three case vignettes were included. The respondents were asked to decide if they would deactivate the PM in the situation given and to indicate the reasons for their decision.

The items of the questionnaire were developed for this survey using a two-step methodology. First, a multidisciplinary research team drafted items that were based on scientific knowledge, a literature survey, and own experiences from clinical practice through an iterative consensus procedure. During the discussions, the research team agreed on items inquiring about the general perception of PM deactivation and secondly on items regarding experiences with, attitudes towards and knowledge of issues in relation to decisions about deactivation of PM. The team focused on clinical problems and omitted differences in dealing with deactivation depending on whether or not the dying process had already started. In addition, three case vignettes were included describing situations where patients with PM dependence ask for withdrawal.

Second, prior to the final application of the questionnaire, cognitive interviews were undertaken with two experienced cardiologists that helped to test problems in feasibility, e.g. comprehensibility of the questions or acceptance. Minor changes in wording were made to the questionnaire according to the results obtained.

Our study targeted cardiologists with experience in pacing and arrhythmia, in particular those who care for patients carrying PMs. We tried for a representative survey by sending our questionnaires to all hospitals in Germany with a cardiology department having experience with PM implantation and follow-up examinations. Questionnaires were sent along with pre-stamped return envelopes to ensure anonymity.

The investigation conforms with the principles outlined in the ‘Declaration of Helsinki’. The study was approved by the local Ethics Committee for Human Research at Philipps-University Marburg, Germany (161–20) and was registered in the German Clinical Trials Register (DRKS00026168).

Statistical analysis was carried out descriptively with SPSS (version 29, 2022).

## Results

Over a period of 4 months out of 288 hospitals 137 (47.6%) responded by sending back at least one questionnaire. In total, we received 247 questionnaires, where 62 hospitals sent one questionnaire, 37 two, 34 three, one four and another five questionnaires.

All of the respondents were cardiologists. Table 1 summarizes sociodemographic data collected.

**Table 1:** Sociodemographic data of respondents answering our questionnaire

	*N* TOTAL	*N* (%)
EXPERIENCE AS CARDIOLOGIST	247	
LESS THAN 5 YRS		39 (15.8)
5–10 YRS		53 (21.5)
11–20 YRS		96 (38.9)
MORE THAN 20 YRS		59 (23.9)
FREQUENCY OF PM CHECK UP	247	
DAILY		172 (69.6)
ONCE WEEKLY		55 (22.3)
RARELY		18 (7.3)
NEVER		2 (0.8)

**Fig. 1:**
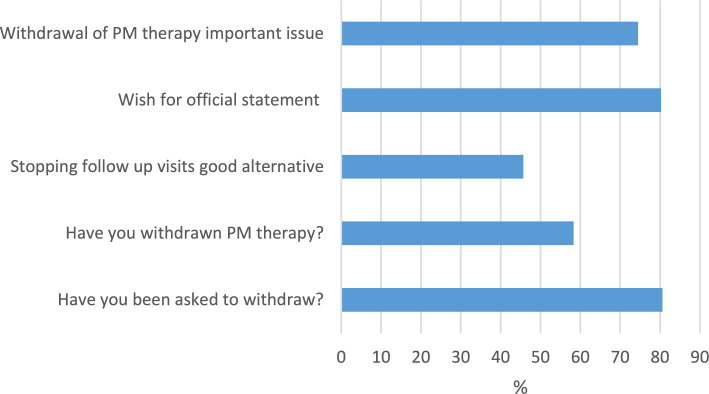
Respondents’ views regarding withdrawal of PM therapy (PM = pacemaker)

Figure 1 shows that most cardiologists have experience with PM withdrawal and consider the issue of PM withdrawal to be important.

When asked under what circumstances the respondent would deactivate a PM upon the patient’s request only 27 (11%) answered that they cannot imagine a situation in which they would withdraw. 189 (77%) answered, that the patient’s EOL should be foreseeable in the near future, and 121 (48%) require the patient to suffer in some way comprehensible. 81 (33%) would ask experienced colleagues for advice. 101 (41%) need a trusting relationship with their patient, and 148 (60%) require there to be a constant wish for deactivation.

When asked, whether the respondents believe that it is possible and correct to fulfil the patient’s wish for withdrawal, 207 (84%) agreed. 141 (57%) argue, that physicians have to respect the patient’s wish for withdrawal and 155 (63%) want to know that the patient is well cared for should problems arise after deactivation.

Amongst the remaining approximately 16%, who would not deactivate the PM, various reasons have been put forward. 31 (13%) fear legal consequences, in 21 (9%) conscience forbids it, 19 (8%) argue, that the PM cannot be deactivated, 3 (1%) don’t as a cardiologist feel responsible for deactivating a PM, 3 (1%) have religious reasons, and 1 (0.4%) believe that the PM is an integral part of the patient’s body.

A few were undecided in that they ticked both ‘yes’ and ‘no’-answers (mainly fearing legal consequences).

Interestingly, about a third—although in principle ready to—are also critical of PM deactivation, arguing that the consequences of withdrawal are often not foreseeable. Also, most PMs would help to lessen symptoms without prolonging life and ask why one should withdraw from a useful therapy. 105 (43%) argue that in the case of pacemaker dependency, deactivation of the pacemaker can lead directly to death, thus providing inadmissible medical assistance in dying.

### Case vignettes

We then asked the respondents to decide whether they would withdraw PM therapy in three case vignettes (see info box).

**Fig. 2:**
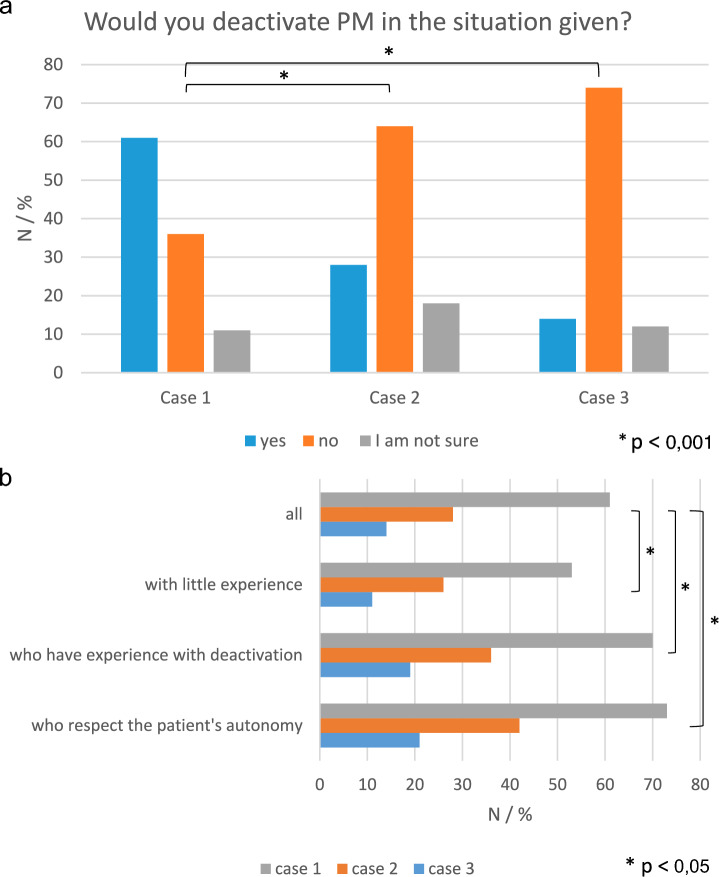
**a** Respondents decisions regarding withdrawal of PM therapy in three case vignettes (see info box for details). **b** Respondents opting for withdrawal of PM therapy in the three case vignettes correlated with their experience or attitudes towards withdrawal

In the first case, 150 (61%) of respondents would withdraw from PM therapy, in the second 70 (28%) and in the third 34 (14%) (Fig. 2a). Reasons given for or against the cardiologists’ decision are listed in Table 2.

**Table 2:** Would you deactivate a pacemaker in the case vignettes described (see info box) and why?

	CASE 1 *N* (%)	CASE 2 *N* (%)	CASE 3 *N* (%)	*P*-VALUE
**POSITIVE ANSWER**	**150 (61)**	**70 (28)**	**34 (14)**	**< 0.001**
YES, BECAUSE I AM CONVINCED THAT I MUST RESPECT THE PATIENT’S AUTONOMY	100 (67)	30 (43)	17 (50)	
YES, BUT I WOULD SEEK ADVICE BEFOREHAND (CLINICAL ETHICS COMITEE, COLLEAGUES, LAWYER)	90 (60)	52 (74)	23 (68)	
**NEGATIVE ANSWER**	**90 (36)**	**159 (64)**	**182 (74)**	** < 0.001**
NO, WITHDRAWAL MIGHT CAUSE UNPLEASANT SYMPTOMS OF, FOR EXAMPLE, LOW-OUTPUT HEART FAILURE	45 (50)	82 (52)	77 (45)	
NO, I HAVE DEACTIVATED PM IN A COMPARABLE SITUATION AND EXPERIENCED THAT THE PATIENT HAD TO SUFFER	1 (1)	4 (3)	8 (4)	
NO, BECAUSE PM DEACTIVATION COULD CAUSE THE PATIENT’S IMMEDIATE DEATH FOR WHICH I WOULD FEEL RESPONSIBLE	50 (54)	93 (59)	125 (69)	
NO, BECAUSE I FEAR LEGAL CONSEQUENCES (= ILLEGAL MEDICAL ASSISTANCE IN DYING)	48 (52)	67 (42)	85 (47)	
**UNKNOWN**	**53 (21.5)**	**70 (28)**	**57 (23)**	**N/A**
I AM UNCERTAIN	27 (60)	45 (64)	30 (53)	
TO DATE, I HAVE NOT BEEN CONFRONTED WITH THIS QUESTION	34 (64)	39 (56)	42 (74)	

When correlating reasons given for each decision in the case vignettes with sociodemographic data or cardiologists’ general views on PM deactivation the following was found: Cardiologists who believe that the patient should be allowed to decide whether a therapy is terminated, or who have experience with deactivating a PM, are significantly more likely to withdraw PM therapy in all cases and significantly less likely to refuse withdrawal (Fig. 2b).

Most interestingly, one (case 1), four (case 2), and eight (case 3) cardiologists saw patients suffer after deactivation of PM in a comparable situation.

### Info Box on case vignettes used in the questionnaire

**Table:** 

We ask you to consider whether you would withdraw the PM in the following cases:	
In all three cases, the patients’ wish for withdrawal is deliberate. The patients have been informed about and have understood the possible consequences. An advance directive exists including the request for withdrawal, which family and friends support	
**1st case vignette:** You are called to the palliative care unit because an 85-year-old patient is dying and urgently requests that his pacemaker be turned off. He is convinced that the PM is keeping him alive unnecessarily. The PM was implanted 20 years ago due to AV block III. The patient is pacemaker dependent *. Life expectancy is believed to be hours, or at most very few days	
**2nd case vignette:** A 60-yr-old patient has recently been diagnosed with metastatic pancreatic cancer. A pacemaker was implanted 2 years ago due to AV block III°. She is pacemaker dependent *. Prognosis is considered to be poor (about 3 months). In this situation, she would like the PM to be deactivated as soon as possible so that she is not kept alive unnecessarily	
**3rd case vignette:** A 75-year-old patient received a pacemaker 5 years ago due to AV block III°. He is pacemaker dependent *. Apart from orthopedic complaints, there are no relevant comorbidities. In his living will he states that he no longer wishes to be kept alive by technical devices when he reaches the age of 75	
Would you withdraw PM therapy at the patient's request?	
*The last 3 routine pacemaker checks showed a ventricular pacing requirement of 100% without junctional or ventricular replacement rhythm during temporary inhibition of ventricular pacing so that complete pacemaker dependence must be assumed.	

## Discussion

Our study provides insights into cardiologists’ views in Germany regarding the withdrawal of PM therapy in general and in PM dependency in particular. Our survey could show that the majority of cardiologists answering our questionnaire are prepared to deactivate a PM upon patients’ request and have done so. But in PM dependency, the willingness to withdraw decreases considerably for fear of causing distressing symptoms, a sense of being responsible for patients possible immediate death, or fear of legal consequences.

In the case vignettes (where patients were PM dependent) the number of respondents ready to withdraw therapy was around 60% in the first case, where the patient is ‘actively dying’, and about 30% in the second, where the patient is terminally ill but has a prognosis of about 3 months. Reasons for refusal given, are either medical (fear of causing distressing symptoms) or personal ones (sense of being responsible for patients’ possible immediate death or fear of legal consequences). In the first case, the patient’s death is imminent due to a terminal illness. If death follows a patient’s competent refusal, withdrawal of an ICD as well as other medical devices such as mechanical ventilation, hemodialysis, intravenous therapy, and percutaneous endoscopic gastrotomy (PEG) feeding is in most western societies ethically and legally permissible [1, 2, 20]. Where withdrawal of PM therapy is concerned, cardiologists have expressed uneasiness for fear of performing medical assistance in dying or assisted suicide [21]. Ethically there is no apparent difference in withdrawal of treatment other than PM [17, 22]. A Heart Rhythm Society (HRS) expert consensus statement [1] has suggested the ethical and legal permissibility of PM deactivation upon patient’s request for the United States of America, emphasizing the patient’s right to decide for him- or herself whether to agree to a therapy or to request it’s discontinuation. In Europe, legal issues regarding the withdrawal of PM therapy are not finally resolved, as is highlighted by the expert consensus statement published by the European Society of Cardiology (ESC) [5].

It can be argued that in the first case vignette PM therapy is futile and that health-related burdens are prolonged unnecessarily. The patient was in a palliative care unit, where unpleasant symptoms could be dealt with should they occur after PM withdrawal. Still, in our survey amongst German cardiologists only 60% of the respondents would deactivate the PM in such a situation. We believe that this finding reflects the need for an official statement resolving legal and practical issues.

In the second case, the patient is terminally ill with a prognosis of 3 months. However, ethical issues apply as stated above. The patient needs to assess the treatment’s value against discomfort and inconveniences associated with his/her illness and its treatment [23]. Clinicians should not overrule patients’ wishes [22] but need to inform patients on possible consequences of withdrawal and have to consider legal issues. The consequences of PM deactivation cannot always be foreseen [1] and data on outcome after withdrawal of PM therapy are rare [24, 25]. In PM dependency, PM therapy may well prolong the dying process in the terminally ill and the patient may ask for withdrawal. In the HRS expert consensus statement mentioned above [1], ethical principles regarding the withdrawal of PM therapy in such a case are discussed based on an algorithm described by Pellegrino [23]. It is emphasized, that only the patient can determine whether the perceived burden of the therapy outweighs the benefit. It is concluded that PM deactivation can be justifiable in the given situation.

The authors concede that the wording in the second case vignette may have been equivocal, as it was not made clear enough that the request for pacemaker deactivation was a consequence of the high disease burden due to the underlying disease.

We believe, that the respondents’ decisions reflect uneasiness regarding the withdrawal of PM therapy in PM-dependent patients as has been reported by others before [11, 16, 26]. This uneasiness might be overcome, should recommendations be issued by professional societies responsible for how to proceed in cases where competent patients ask for the withdrawal of PM therapy. Such detailed recommendations are currently lacking in Europe. The 2021 European guidelines on PM therapy include one sentence, only, saying that competent patients have the right to refuse PM therapy [5]. An older guideline from 2010 focuses on the deactivation of ICDs in patients with an irreversible or terminal illness [1, 2, 23], but not PM withdrawal.

In the third case, where the patient is apparently healthy apart from carrying a PM, only a few of the respondents would deactivate the PM: On principle, the same arguments apply as mentioned above. The patient’s reasons for withdrawal have to be heard and discussed, especially as patients with a PM wish to do so [27, 28]. In the case vignette, no such reasons are given. It is not known if the patient wants to remove a futile treatment or to hasten death, in that he requests a form of physician-assisted suicide. If the latter is the case, the patient should be counselled regarding the legal possibility of assisted suicide, since the imponderabilites of PM withdrawal are such that it cannot be recommended, and alternative methods are available. Only if the reasons behind the request for withdrawal are given a solution can be sought. It is therefore understandable, that most of the respondents refuse to deactivate the PM in the case vignette given.

Interestingly, one (case 1), four (case 2), and eight (case 3) cardiologists saw patients suffer after deactivation of PM in a comparable situation and would therefore deny withdrawal in the case vignettes presented. These experiences again call for a consensus statement by cardiologic societies of each European country comprising ethical, legal and practical issues of PM withdrawal to support cardiologists in these difficult situations. Not surprisingly, the majority of respondents in our survey opted for an official statement of the German cardiologic society.

The discrepancy between the willingness to deactivate in principle and actual deactivation in individual cases, as shown in the case vignettes, was reported by others as well [15, 16, 30]. The same applies to the recommendation of conversations regarding the withdrawal of ICD therapy. When a terminal illness is diagnosed, information on the possibility of ICD withdrawal should be offered to avoid unnecessary painful shocks in the dying process [15, 16, 29, 30, 31]. As has been mentioned before, PM withdrawal should be treated accordingly.

Our survey could also show, that cardiologists faced with a patient’s desire to withdraw PM therapy, have practical issues in mind. The HRS consensus statement [1] clearly states that it must be clarified beforehand how device deactivation can effectively be put into practice. In the said statement, cardiology departments implanting CIEDs are required to ensure that personnel and facilities are provided to enable patients to have their CIEDs deactivated at their request.

### Limitations

Apart from the known general limitations of surveys, such as deductive assumptions in the conception of the questions, the study has several additional limitations. The group under study is not representative for cardiologists in general. Cardiologists were recruited by sending the questionnaire to all hospitals in Germany with a cardiologic department registered for PM implantations, almost half (47.6%) of them responded. Cardiologists working in outpatient clinics or practices were not included. Cardiologists were recruited during the COVID-19 pandemic. Since the questionnaire was sent in paper form, the impact of the pandemic will not have been relevant.

## Conclusion

Our survey shows that the majority of cardiologists answering our questionnaire are prepared to deactivate a PM upon patients’ request and have done so. In PM dependency the willingness to withdraw decreases for fear of causing distressing symptoms, sense of being responsible for the patients possible immediate death, or fear of legal consequences. The results of the survey suggest that official statements of the cardiologic societies in Europe are warranted to clarify ethical, legal, and practical aspects of PM withdrawal. Especially because in an aging population the number of patients with PMs and subsequently requests for withdrawal will increase in number.

## Data Availability

The datasets used are available from the corresponding author upon reasonable request.

## Abbreviations

AVB: Atrioventricular block
EOL: End of life
ESC: European Society of Cardiology
EHRA: European Heart Rhythm Association
HRS: Heart Rhythm Society
ICD: Implantable cardioverter defibrillator
PM: Pacemaker
SND: Sinunodal disease

## References

[1] Lampert R, Hayes DL, Annas GJ, Farley MA, Goldstein NE, Hamilton RM et al (2010) HRS expert consensus statement on the management of cardiovascular implantable electronic devices (CIEDs) in patients nearing end of life or requesting withdrawal of therapy. Heart Rhythm 7(7):1008–1026 20471915 10.1016/j.hrthm.2010.04.033

[2] Padeletti L, Arnar DO, Boncinelli L, Brachman J, Camm JA, Daubert JC et al (2010) EHRA expert consensus statement on the management of cardiovascular implantable electronic devices in patients nearing end of life or requesting withdrawal of therapy. Europace 12(10):1480–1489 20675674 10.1093/europace/euq275

[3] Waltenberger J, Schöne-Seifert B, Friedrich DR, Alt-Epping B, Bestehorn M, Dutzmann J et al (2017) Verantwortlicher umgang mit ICDs. Kardiologe 11(5):383–397

[4] Schneider J (2024) Deaktivierung von Implantaten am Lebensende. 1st ed. Mohr Siebeck

[5] Glikson M, Nielsen JC, Kronborg MB, Michowitz Y, Auricchio A, Barbash IM et al (2021) 2021 ESC guidelines on cardiac pacing and cardiac resynchronization therapy. Eur Heart J 42(35):3427–3520 34455430 10.1093/eurheartj/ehab364

[6] Kusumoto FM, Schoenfeld MH, Barrett C, Edgerton JR, Ellenbogen KA, Gold MR et al (2019) 2018 ACC/AHA/HRS guideline on the evaluation and management of patients with bradycardia and cardiac conduction delay. J Am Coll Cardiol 74(7):e51–e156 30412709 10.1016/j.jacc.2018.10.044

[7] Lamas GA, Orav EJ, Stambler BS, Ellenbogen KA, Sgarbossa EB, Huang SK et al (1998) Quality of life and clinical outcomes in elderly patients treated with ventricular pacing as compared with dual-chamber pacing. Pacemaker selection in the elderly investigators. N Engl J Med 338(16):1097–1104 9545357 10.1056/NEJM199804163381602

[8] Majewski JP, Lelakowski J (2018) Pacemaker dependency: How should it be defined? Europace 20(10):1708 29518191 10.1093/europace/euy010

[9] Pitcher D, Soar J, Hogg K, Linker N, Chapman S, Beattie JM et al (2016) Cardiovascular implanted electronic devices in people towards the end of life, during cardiopulmonary resuscitation and after death: guidance from the resuscitation council (UK), British cardiovascular society and national council for palliative care. Heart 102(Suppl 7):A1–A17 27277710 10.1136/heartjnl-2016-309721

[10] Grimm W, Grimm K, Greene B, Parahuleva M (2021) Predictors of pacing-dependency in patients with cardiovascular implantable electronic devices. Cardiol J 28(3):423–430 31489608 10.5603/CJ.a2019.0088 PMC8169185

[11] Goldstein N, Bradley E, Zeidman J, Mehta D, Morrison RS (2009) Barriers to conversations about deactivation of implantable defibrillators in seriously ill patients: results of a nationwide survey comparing cardiology specialists to primary care physicians. J Am Coll Cardiol 54(4):371–373 19608038 10.1016/j.jacc.2009.04.030 PMC3059516

[12] Kapa S, Mueller PS, Hayes DL, Asirvatham SJ (2010) Perspectives on withdrawing pacemaker and implantable cardioverter-defibrillator therapies at end of life: results of a survey of medical and legal professionals and patients. Mayo Clin Proc 85(11):981–990 20843982 10.4065/mcp.2010.0431 PMC2966361

[13] Kelley AS, Reid MC, Miller DH, Fins JJ, Lachs MS (2009) Implantable cardioverter-defibrillator deactivation at the end of life: a physician survey. Am Heart J 157(4):702–8.e1 19332199 10.1016/j.ahj.2008.12.011

[14] Marinskis G, van Erven L (2010) Deactivation of implanted cardioverter-defibrillators at the end of life: results of the EHRA survey. Europace 12(8):1176–1177 20663788 10.1093/europace/euq272

[15] Mueller PS (2010) Clinicians' views regarding deactivation of cardiovascular implantable electronic devices in seriously ill patients. Heart Rhythm 7(11):1543–1544. Available from: URL: https://pubmed.ncbi.nlm.nih.gov/20816871/10.1016/j.hrthm.2010.08.020 20816871

[16] Mueller PS, Jenkins SM, Bramstedt KA, Hayes DL (2008) Deactivating implanted cardiac devices in terminally ill patients: practices and attitudes. Pacing Clin Electrophysiol 31(5):560–568 18439169 10.1111/j.1540-8159.2008.01041.x

[17] Bevins MB (2011) The ethics of pacemaker deactivation in terminally ill patients. J Pain Symptom Manage 41(6):1106–1110 21621131 10.1016/j.jpainsymman.2011.03.003

[18] Hui D, Nooruddin Z, Didwaniya N, Dev R, de La Cruz M, Kim SH et al (2014) Concepts and definitions for “actively dying,” “end of life,” “terminally ill,” “terminal care,” and “transition of care”: a systematic review. J Pain Symptom Manage 47(1):77–89 23796586 10.1016/j.jpainsymman.2013.02.021 PMC3870193

[19] White N, Reid F, Harries P, Harris AJL, Minton O, McGowan C et al (2019) The (un)availability of prognostic information in the last days of life: a prospective observational study. BMJ Open 9(7):e030736 31292186 10.1136/bmjopen-2019-030736 PMC6624101

[20] Gedge E, Giacomini M, Cook D (2007) Withholding and withdrawing life support in critical care settings: ethical issues concerning consent. J Med Ethics 33(4):215–218 17400619 10.1136/jme.2006.017038 PMC2652778

[21] Kramer DB, Kesselheim AS, Brock DW, Maisel WH (2010) Ethical and legal views of physicians regarding deactivation of cardiac implantable electrical devices: a quantitative assessment. Heart Rhythm 7(11):1537–1542 20650332 10.1016/j.hrthm.2010.07.018 PMC3001282

[22] Zellner RA, Aulisio MP, Lewis WR (2009) Should implantable cardioverter-defibrillators and permanent pacemakers in patients with terminal illness be deactivated? Deactivating permanent pacemaker in patients with terminalillness. Patient autonomy is paramount. Circ Arrhythm Electrophysiol 2(3):340–344 (**discussion 340**) 19808485 10.1161/CIRCEP.109.848523

[23] Pellegrino ED (2000) Decisions to withdraw life-sustaining treatment: a moral algorithm. JAMA 283(8):1065–1067 10697071 10.1001/jama.283.8.1065

[24] Buchhalter LC, Ottenberg AL, Webster TL, Swetz KM, Hayes DL, Mueller PS (2014) Features and outcomes of patients who underwent cardiac device deactivation. JAMA Intern Med 174(1):80–85 24276835 10.1001/jamainternmed.2013.11564 PMC4266591

[25] Lewis WR, Luebke DL, Johnson NJ, Harrington MD, Costantini O, Aulisio MP (2006) Withdrawing implantable defibrillator shock therapy in terminally ill patients. Am J Med 119(10):892–896 17000222 10.1016/j.amjmed.2006.01.017

[26] Beca JP, Rosselot E, Asenjo R, Anguita V, Quevedo R (2009) Deactivating cardiac pacemakers and implantable cardioverter defibrillators in terminally ill patients. Camb Q Healthc Ethics 18(3):236–240 19460224 10.1017/S0963180109090380

[27] Portig I, Karaaslan E, Hofacker E, Volberg C, Seifart C (2023) Patients’ perspective on termination of pacemaker therapy—a cross-sectional anonymous survey among patients carrying a pacemaker in Germany. Healthcare 11(21):2896 37958040 10.3390/healthcare11212896 PMC10649284

[28] Rodríguez-Prat A, Monforte-Royo C, Porta-Sales J, Escribano X, Balaguer A (2016) Patient perspectives of dignity, autonomy and control at the end of life: systematic review and meta-ethnography. PLoS ONE 11(3):e0151435 27010323 10.1371/journal.pone.0151435 PMC4806874

[29] Tischer T, Bebersdorf A, Albrecht C, Manhart J, Büttner A, Öner A et al (2020) Deactivation of cardiovascular implantable electronic devices in patients nearing end of life: Reality or only recommendation? Herz 45(Suppl 1):123–129 31312871 10.1007/s00059-019-4836-1

[30] Goldstein NE, Lampert R, Bradley E, Lynn J, Krumholz HM (2004) Management of implantable cardioverter defibrillators in end-of-life care. Ann Intern Med 141(11):835–838 15583224 10.7326/0003-4819-141-11-200412070-00006

[31] Standing H, Thomson RG, Flynn D, Hughes J, Joyce K, Lobban T et al (2021) “You can’t start a car when there’s no petrol left”: a qualitative study of patient, family and clinician perspectives on implantable cardioverter defibrillator deactivation. BMJ Open 11(7):e048024 34230020 10.1136/bmjopen-2020-048024 PMC8261879

